# Inflammatory indices obtained from routine blood tests show an inflammatory state associated with disease progression in engineered stone silicosis patients

**DOI:** 10.1038/s41598-022-11926-x

**Published:** 2022-05-17

**Authors:** Alejandro García-Núñez, Gema Jiménez-Gómez, Antonio Hidalgo-Molina, Juan Antonio Córdoba-Doña, Antonio León-Jiménez, Antonio Campos-Caro

**Affiliations:** 1 Biomedical Research and Innovation Institute of Cadiz (INiBICA), 11009 Cádiz, Spain; 2 Research Unit, Puerta del Mar University Hospital, 11009 Cádiz, Spain; 3 Pulmonology, Allergy and Thoracic Surgery Department, Puerta del Mar University Hospital, 11009 Cádiz, Spain; 4 Department of Preventive Medicine and Public Health, Jerez University Hospital, 11407 Jerez de la Frontera, Spain; 5 Genetics Area, Biomedicine, Biotechnology and Public Health Department, School of Marine and Environmental Sciences, University of Cadiz, 11510 Cádiz, Spain

**Keywords:** Respiratory tract diseases, Predictive markers, Prognostic markers

## Abstract

Patients with silicosis caused by occupational exposure to engineered stone (ES) present a rapid progression from simple silicosis (SS) to progressive massive fibrosis (PMF). Patient classification follows international rules based on radiology and high-resolution computed tomography (HRCT), but limited studies, if any, have explored biomarkers from routine clinical tests that can be used as predictors of disease status. Our objective was thus to investigate circulating biomarker levels and systemic inflammatory indices in ES silicosis patients whose exposure to ES dust ended several years ago. Ninety-one adult men, ex-workers in the manufacturing of ES, 53 diagnosed with SS and 38 with PMF, and 22 healthy male volunteers (HC) as controls not exposed to ES dust, were recruited. The following circulating levels of biomarkers like lactate dehydrogenase (LDH), angiotensin-converting-enzyme (ACE), protein C reactive (PCR), rheumatoid factor, alkaline phosphatase and fibrinogen were obtained from clinical reports after being measured from blood samples. As biochemical markers, only LDH (HC = 262 ± 48.1; SS = 315.4 ± 65.4; PMF = 337.6 ± 79.3 U/L), ACE (HC = 43.1 ± 18.4; SS = 78.2 ± 27.2; PMF = 86.1 ± 23.7 U/L) and fibrinogen (HC = 182.3 ± 49.1; SS = 212.2 ± 43.5; PMF = 256 ± 77.3 U/L) levels showed a significant sequential increase, not been observed for the rest of biomarkers, in the HC → SS → PMF direction. Moreover, several systemic inflammation indices neutrophil-to-lymphocyte ratio (NLR), lymphocyte-to-monocyte ratio (LMR), platelet-to-lymphocyte ratio (PLR), systemic inflammation response index (SIRI), systemic immune-inflammation index (SII), aggregate index of systemic inflammation (AISI) derived from whole blood cell counts showed significant differences between the HC, SS and PMF groups. All these biomarkers were analyzed using receiver operating characteristic (ROC) curves, and the results provided moderately high sensitivity and specificity for discriminating between ES silicosis patient groups and healthy controls. Our study reveals that some inflammatory biomarkers, easily available from routine blood analysis, are present in ES silicosis patients even several years after cessation of exposure to ES silica dust and they could help to know the progression of the disease.

## Introduction

Silicosis is an occupational disease cataloged into the group of interstitial lung diseases due to the continuous inhalation of free crystalline silica that can lead to incapacitating lung fibrosis and respiratory failure^1^. Known for centuries as a disease related to industry and mining, this disease has re-emerged with the use of new artificial compounds of silica in construction. These artificial agglomerates of silica, engineered stone (ES) or artificial stone (AS), have seen extensive use in recent years in the field of construction in the manufacturing of kitchen and bathroom countertops^2^. Engineered Stone is a composite material fabricated with finely crushed natural rocks with high content in silica, assembled with polymeric resins as a binder, and in general, adding different pigments like metals. Usually, ES is composed of 10–15% of resins and the rest is silica, cristobalite or quartz in different concentrations^3^. Reports of a significant number of affected workers initially came from Israel^4^ and Spain^5^. To date, ES silicosis has been identified in many countries, such as the United States^6^, China^7^ and Australia^8^, and in some of them, it is considered an occupational epidemic.

ES-related silicosis is characterized by a short latency period, extensive pulmonary damage, and its occurrence in young workers^9^. In addition, the progression from simple chronic silicosis (SS) to progressive massive fibrosis (PMF) of this entity continues even after cessation of exposure to silica. In this way, up to 56% of patients show progression, and 37% are diagnosed with PMF after a mean follow-up of 4.01 ± 2.1 years^10^. The mechanisms of the increased aggressiveness of ES silicosis are not totally known, but some authors have pointed to the role of high levels of exposure generated over a short period of time in certain tasks (cutting, polishing, etc.)^9^. Also, the higher reactivity in free radical production correlates with the large amount of transition metals ions contained in ES dusts^11^. Finally, it is worth mentioning the potential effect of resins coating the crystalline silica, which protect the surface radicals for a limited time, so they are not annihilated before reaching lung tissues^12^.

The diagnosis and progression of the disease are generally based on ILO classification by chest radiography^13^ and ICOERD classification by high-resolution computed tomography (HRCT)^14^. However, although those standards are the key to patient classification, other parameters to establish more precise discrimination between borderline patients and healthy people could be a useful tool for clinicians.

The role of the immune system in the pathogenesis of this disease is not fully known^15,16^, and indicators of systemic inflammation in routine blood tests in silicosis patients have been sparsely investigated. At the beginning of the twentieth century, peripheral white blood cell (WBC) count was already reported as a diagnostic indicator of some diseases, but it was not until the last two decades that differential WBC subsets including neutrophils, lymphocytes, monocytes, platelets, eosinophils and basophils have emerged as cheap and easily measurable biomarkers. Neutrophils and monocytes are essential components of the innate immune response, lymphocytes are involved in adaptive immune response and platelets also play an important role, in addition to hemostasis, coagulation and angiogenesis, in innate immunity and inflammatory reaction. WBC count and its derived indicators such as the neutrophil-to-lymphocyte ratio (NLR) based on the coexistence of lymphopenia and leukocytosis in the initial inflammatory response^17,18^, lymphocyte-to-monocyte ratio (LMR) can reflect the body’s immune status, and its decrease indicates host immune dysfunction^19^, platelet-to-lymphocyte ratio (PLR) is an inflammatory marker of immune-mediated, metabolic, prothrombotic, and neoplastic diseases^20^, have been considered as predictors of severity and prognosis of the disease although, by combining several of these parameters, the prediction can be greatly improved. Then, others more inclusive ratios such as: (i) neutrophil x monocyte-to-lymphocyte ratio (systemic inflammation response index or SIRI), which has a stronger stability than NLR and an elevated SIRI suggests increased inflammation and reduced immune response in patients^21,22^; (ii) neutrophil x platelet-to-lymphocyte ratio (systemic immune-inflammation index or SII) with poor prognosis when augmented^23,24^ and (iii) neutrophil x monocyte x platelet-to-lymphocyte ratio (aggregate index of systemic inflammation or AISI)^25^ have been proposed as cheap and easily measurable biomarkers or indicators to improve the diagnosis/prognosis in numerous and different pathologies. All these indices can be used as indicators of inflammatory state and severity to evaluate the diagnosis and the clinical prognosis of patients with ES silicosis because, although clinical and radiologic factors may assist in predicting the likelihood of complications and mortality from ES silicosis, evaluating the prognosis of these patients remains challenging.

Recently, some routine hematological and biochemical tests have shown significant alterations in new combined blood cell count indices of inflammation in idiopathic pulmonary fibrosis (IPF)^26^ and connective tissue diseases with pulmonary involvement^27^. Specifically, several works with NLR and PLR have been reported in silicosis disease^28–30^, showing that these routine indices and parameters could detect a chronic inflammatory status and help identify patients who may evolve to more severe forms of these diseases; however, no reports have been published in ES silicosis patients, and even less is known about these parameters after prolonged exposure cessation.

Thus, the aim of this cross-sectional analytical study is to determine whether ES silicosis patients, even years after exposure cessation, show changes in biochemical markers and in blood cell count-derived inflammation indices obtained in routine blood tests. Moreover, we analyzed some independent prognostic risk factors and their relationship with the clinical parameters.

## Results

### Characteristics of the study subjects and respiratory clinical parameters

A total of 91 patients with silicosis agreed to participate in the study, of whom 53 had simple chronic silicosis (SS) and 38 had progressive massive fibrosis (PMF). All subjects studied were males, and their sociodemographic data are shown in Table 1. The mean age, starting age and duration of exposure to engineered stone dust were all similar, without significant differences between the groups studied. A healthy control group (HC) not exposed to silica dust was also studied. Functional parameters measured in the patient groups are also shown in Table 1. Significant differences were observed when the mean values for FEV_1_, FVC, DLCO and FEV_1_/FVC between the SS and PMF groups were compared. Data from the modified Medical Research Council scale of dyspnea (mMRC Dyspnea) did not present differences between groups (data not shown).

**Table 1:** Sociodemographic data of participants and pulmonary function values of patients with SS and PMF.

	HC (n = 22)	SS (n = 53)	PMF (n = 38)	*P*
Age*	36.4 ± 8.3	40.1 ± 7.7	41 ± 6.2	0.052^+^
Starting Exposure Age*	–	21.2 ± 7.4	21.4 ± 4.3	0.142^++^
Duration of Exposure*	–	13.1 ± 6.7	13.3 ± 6.1	0.968^++^
Years from cessation of exposure to blood extraction*	–	6.4 ± 2.7	7.3 ± 2.5	0.058^++^
Smoking status**				0.099^+++^
Non-Smoker	15 (65.2)	22 (41.5)	15 (39.5)	
Ex-Smoker	5 (21.7)	26 (49.1)	21 (55.3)	
Smoker	3 (13)	5 (9.4)	2 (5.3)	
FEV_1_ (mL)*	nd	3,386 ± 647	2,961 ± 631	0.003
FEV_1_ (%)*	nd	87.8 ± 14	76.5 ± 14.8	< 0.0001
FVC (mL)*	nd	4,341 ± 748	3,961 ± 783	0.022
FVC (%)*	nd	90.1 ± 13.3	82.3 ± 14.8	0.01
FEV_1_/FVC*	nd	0.77 ± 0.05	0.74 ± 0.07	0.009
DLCO (mmol/min/kPa)*	nd	9.2 ± 1.7	8.3 ± 1.4	0.006
DLCO (%)*	nd	85.4 ± 14.8	77.6 ± 14	0.014

Forced expiratory volume in 1 s (FEV1), forced vital capacity (FVC), diffusing capacity of lung for carbon monoxide (DLCO). *Mean ± standard deviation. **Number of cases (percentage). ^+^ANOVA F-test, ^++^Mann–Whitney-*U* test, ^+++^χ^2^ test, ^++++^Student´s t-test; *nd* not determined.

### Biochemical and hematological markers in blood from ES silicosis patients

**Figure 1:**
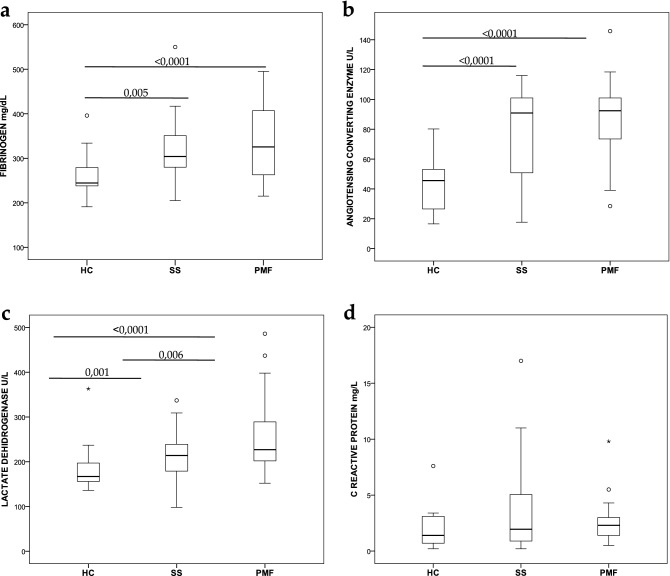
Box plots showing results from serum circulating markers. (**a**) Fibrinogen (mg/dL), (**b**) ACE (U/L), (**c**). LDH (U/L), (**d**) CRP (mg/L). Boxes show interquartile ranges. Lines in the boxes correspond to medians, and bars represent the lowest and highest values. Circles represent outliers. Student’s t-test in (**a**) and the Mann–Whitney *U*-test in (**b**–**d**) were applied. *p* values with significant differences between groups are indicated by horizontal lines.

Usually, routine clinical tests include a panel of molecular analyses used as functional markers of health disorders. For this reason, to find some differences between the three groups studied (HC, SS and PMF), we decided to analyze the main biochemical parameters obtained after peripheral blood extraction. Figure 1 shows some of the analytical markers analyzed. Figure 1A reveals a significant increase in peripheral blood circulating fibrinogen levels between the HC group (262 ± 48.1) and either of the silicotic groups, SS 1B). A significant increase was observed in patients’ ACE levels (78.2 ± 27.2 in SS and 86.1 ± 23.7 in PMF) compared to controls (43.1 ± 18.4), but there were no differences between the two groups of patients. Nevertheless, as shown in Fig. 1C, lactate dehydrogenase (LDH) level analysis displayed a significant progressive increase between all groups studied, HC (182.3 ± 49.1), SS (212.2 ± 43.5) and PMF (256 ± 77.3). As an example of another proinflammatory marker included in this study that did not reveal any differences between controls and neither of the two groups of patients, was C-reactive protein (CRP) (Fig. 1D) but other markers, i.e., rheumatoid factor and alkaline phosphatase, did not show differences either (data not shown).

### Leukocyte subpopulations and systemic inflammatory indices

The total leukocyte count obtained from the routine analysis did not show differences between HCs and silicosis patients (Table 2). However, several significant differences were observed when leukocyte subsets were compared. Therefore, the monocyte count showed a trend of increasing from HC → SS → PMF, but only significant differences between HC-PMF and SS-PMF were observed. Clear significant lymphocytopenia was observed in both silicotic patient groups compared to the HC group but not between the silicotic groups. Neutrophils and platelets did not present a significant difference between either of the groups studied, although an increasing trend was also observed for neutrophil counts in the SS and PMF groups.

**Table 2:** White blood cell counts and leukocyte subset indices.

	HC (22)	SS (53)	PMF (38)	*P*
Leukocytes*	6.3 ± 1.4	6.1 ± 1.7	6.4 ± 1.4	0.198^a^
Monocytes*	0.5 ± 0.1	0.56 ± 0.2	0.6 ± 0.2	0.012^b^ HC vs. SS; = ns^c^ HC vs. PMF; = 0.008^c^ SS vs. PMF; = 0.032^c^
Lymphocytes*	2.2 ± 0.6	1.7 ± 0.5	1.6 ± 0.4	0.001^a^ HC vs. SS; = 0.001^d^ HC vs. PMF; < 0.0001^c^ SS vs. PMF; = ns^d^
Neutrophils*	3.4 ± 0.9	3.7 ± 1.4	4 ± 1.2	0.071^a^
Platelets*	222.9 ± 40.6	239.8 ± 59.7	242.5 ± 40.6	0.334^b^
NLR	1.6 ± 0.5	2.4 ± 1	2.7 ± 1.5	< 0.0001^a^ HC vs. SS; = 0.002^d^ HC vs. PMF; < 0.0001^d^ SS vs. PMF; = ns^d^
PLR	108.3 ± 37.1	154.5 ± 54.8	160.5 ± 53.5	< 0.0001^a^ HC vs. SS; = 0.001^c^ HC vs. PMF; < 0.0001^d^ SS vs. PMF; ns^d^
LMR	4.5 ± 1.5	3.1 ± 0.9	2.7 ± 1	< 0.0001^a^ HC vs. SS; < 0.0001^c^ HC vs. PMF; < 0.0001^d^ SS vs. PMF; = 0.044^d^
SII	369.5 ± 157	567.3 ± 290.7	668.9 ± 424.7	< 0.0001^a^ HC vs. SS; = 0.002^d^ HC vs. PMF; < 0.0001^d^ SS vs. PMF; ns^d^
SIRI	0.8 ± 0.4	1.3 ± 0.7	1.8 ± 1.2	< 0.0001^a^ HC vs. SS; = 0.002^d^ HC vs. PMF; < 0.0001^d^ SS vs. PMF; = 0.036^d^
AISI	191.5 ± 109.6	330.8 ± 219.8	447.1 ± 314.1	< 0.0001a HC vs. SS; = 0.006^d^ HC vs. PMF; < 0.0001^d^ SS vs. PMF; = 0.044^d^

NLR (neutrophil/lymphocyte ratio), PLR (platelet/lymphocyte ratio), LMR (lymphocyte/monocyte ratio), SII (neutrophil x platelet/lymphocyte ratio), SIRI (neutrophil x monocyte/lymphocyte ratio) and AISI (neutrophil x monocyte x platelet/lymphocyte ratio). *(× 10^3^ cell/µl). Data represent mean ± standard deviation. ^a^Kruskal–Wallis test, ^b^ANOVA, ^c^Student´s T-test, ^d^Mann–Whitney-*U* test. *P*, *p* value; *ns* non-significant.

Additionally, some combined ratios of the leukocyte count, such as NLR, PLR, LMR, SII, SIRI and AISI, were calculated as inflammation indices to determine the inflammatory status of silicotic patients (Table 2 and supplementary Fig. S1). All analyzed ratios showed significant differences between the HC and silicotic groups, and furthermore, the LMR, SIRI and AISI indices showed significant differences between SS and PMF patients.

### Association of biochemical, cellular and functional parameters with ES silicosis status

To evaluate whether the biochemical and/or cellular markers studied could be used to help classify silicotic patients in the SS or PMF groups, we performed receiver operating characteristic (ROC) curve analysis. The ROC curve is one of the most popular graphical tool for evaluating the diagnostic power of a biomarker in relation to a gold standard procedure. This is a graphical representation of the relationship between sensitivity (proportion of true positives) and 1-specificity (proportion of false positives) of a given biomarker to be used in diagnosis^31–33^. The area under the ROC curve (AUC) is a single number that offers an estimation of the probability of correctly classifying a random subject, i.e. an AUC of 0.85 indicates an 85% likelihood of correctly classifying the subject. An AUC of 1.0 represents a test with perfect discrimination, while AUC of 0.5 represents a test result no better than if determined by chance alone.

**Figure 2:**
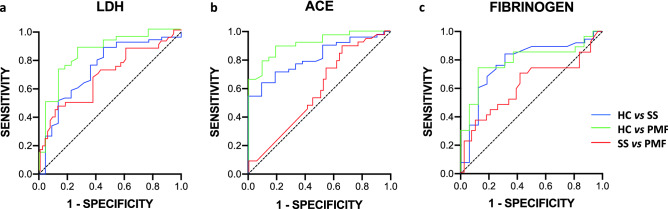
ROC curves using LDH, ACE and fibrinogen biochemical markers for predictive ES silicosis status between studied groups. (**a**) LDH, (**b**) ACE and (**c**) Fibrinogen.

Initially, as expected, just considering the biomarkers fibrinogen, ACE and LDH independently, a clear discrimination between HC and any of the patient groups was observed (Fig. 2). All data regarding the area under the ROC curve (AUC) are depicted in Table 3. However, these biomarkers did not serve to discriminate between the two groups of patients, SS and PMF (Fig. 2, Table 3).

**Table 3:** AUC data from single biochemical marker/systemic inflammation index ROC curves.

	Biomarker/Index	AUC	95% CI	*P*
HC vs. SS	LDH	0.7431	0.6161–0.8701	0.0009
	ACE	0.8190	0.7248–0.9131	< 0.0001
	Fibrinogen	0.7780	0.6358–0.9201	0.0014
	NLR	0.7382	0.6243–0.8521	0.0011
	PLR	0.7532	0.6379–0.8685	0.0005
	LMR	0.767	0.6449–0.8891	0.0003
	SII	0.7338	0.6138–0.8537	0.0014
	SIRI	0.7377	0.6108–0.8646	0.0014
	AISI	0.7143	0.5818–0.8468	0.0039
HC vs. PMF	LDH	0.8374	0.7291–0.9457	< 0.0001
	ACE	0.9142	0.8446–0.9838	< 0.0001
	Fibrinogen	0.7870	0.6466–0.9275	0.0018
	NLR	0.8281	0.7234–0.9328	< 0.0001
	PLR	0.8100	0.6916–0.9285	< 0.0001
	LMR	0.8535	0.7531–0.9539	< 0.0001
	SII	0.8054	0.6922–0.9185	< 0.0001
	SIRI	0.8138	0.7060–0.9215	< 0.0001
	AISI	0.8059	0.6951–0.9167	0.0001
SS vs. PMF	LDH	0.6811	0.5689–0.07933	0.0028
	ACE	0.5747	0.4570–0.6924	0.2258
	Fibrinogen	0.6179	0.4713–0.7645	0.1072
	NLR	0.5595	0.4438–0.6753	0.3254
	PLR	0.5385	0.4224–0.6546	0.5251
	LMR	0.6277	0.5133–0.7422	0.0348
	SII	0.5733	0.4574–0.6891	0.2261
	SIRI	0.6264	0.5102–0.7426	0.0368
	AISI	0.6227	0.5075–0.7379	0.0426

AUC, area under the ROC curve; 95% CI, 95% confidence interval; *LDH* lactate dehydrogenase; *ACE* angiotensin convertase enzyme; NLR (neutrophil/lymphocyte ratio), PLR (platelet/lymphocyte ratio), LMR (lymphocyte/monocyte ratio), SII (neutrophil x platelet/lymphocyte ratio), SIRI (neutrophil x monocyte/lymphocyte ratio) and AISI (neutrophil x monocyte x platelet/lymphocyte ratio). 95% CI, 95% confidence interval; *P*, *p value.*

**Figure 3:**
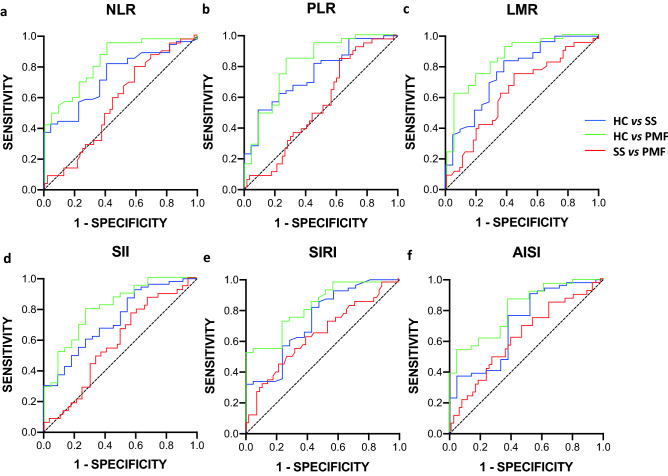
ROC curves using systemic inflammation indices for predictive ES silicosis status between studied groups. (**a**) NLR (neutrophil/lymphocyte ratio), (**b**) PLR (platelet/lymphocyte ratio), (**c**) LMR (lymphocyte/monocyte ratio), (**d**) SII (neutrophil x platelet/lymphocyte ratio), (**e**) SIRI (neutrophil x monocyte/lymphocyte ratio), (**f**) AISI (neutrophil x monocyte x platelet/lymphocyte ratio).

In the same way, ROC curves were carried out for all systemic inflammation indices previously analyzed (Fig. 3), and the AUC is shown in Table 4. The ROC curves proved that all inflammatory indices have some usefulness for evaluating the severity of ES silicosis patients, more obviously for PMF, but with non-negligible values it is also highly significant for SS diagnosis. However, significantly poor values were obtained when assessing AUCs between SS and PMF.

**Table 4:** Combined AUC ROC curve data.

	Biomarkers	AUC	95% CI	*P*
HC vs. SS	LAF	0.8767	0.7692 to 0.9842	< 0.0001
	LAF-SIRI	0.8889	0.7779 to 0.9999	< 0.0001
	LAF-SIRI-FRT	nd	nd	nd
HC vs. PMF	LAF	0.9444	0.8793 to 1.000	< 0.0001
	LAF-SIRI	1.000	1.000	< 0.0001
	LAF-SIRI-FRT	nd	nd	nd
SS vs. PMF	LAF	0.7263	0.5981 to 0.8546	0.0022
	LAF-SIRI	0.7891	0.6765 to 0.9017	< 0.0001
	LAF-SIRI-FRT	0.9143	0.8410 to 0.9876	< 0.0001

LAF, combined LDH-ACE-fibrinogen; LAF-SIRI, LAF plus SIRI index; LAF-SIRI-FRT, LAF-SIRI plus FVC and DLCO; AUC, area under the ROC curve; 95% CI, 95% confidence interval; *P*, *p* value.

**Figure 4:**
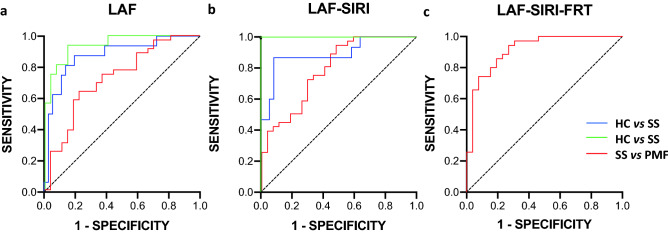
Combined ROC curves for a multiple logistic regression analysis with several markers. (**a**) LAF (LDH-ACE-fibrinogen), (**b**) LAF-SIRI, (**c**) LAF-SIRI-FRT (LAF-SIRI-FVC-DLCO). Note that LAF-SIRI-FRT could only be tested between patient comparisons.

Based on the results and considering that single markers are not sufficiently robust for diagnosis or for stratification of the diseases, combined markers were used to create new ROC curves. Therefore, several biochemical and inflammatory indices and even functional markers were used in combined ROCs to evaluate whether they can be used as effective indicators to discriminate between ES silicosis patients or healthy people. In Fig. 4, several combined ROC curves are shown, including the three biochemical markers analyzed above LDH, ACE and fibrinogen (indicated as LAF), the same biochemical markers plus the inflammation index SIRI (indicated as LAF-SIRI) and finally, all markers plus the functional respiratory tests FVC and DLCO (indicated as LAF-SIRI-FRT). The analysis of the combined ROC curves is shown in Table 4.

## Discussion

Compared to natural stone-associated silicosis, ES-associated silicosis is characterized by short latency, rapid radiological progression, accelerated decline in lung function and high mortality^7^. Moreover, a considerable proportion of patients with ES silicosis progress rapidly even following cessation of exposure. After an average follow-up of 4 years, 37.7% of patients had progressed to PMF, and 25% experienced a decline in FVC > 157 mL per year^10^.

The molecular mechanism that induces silicosis is not totally understood despite the accumulated information on the matter^1,2,34^. Inhalation of crystalline silica dust produce a deposition of these particles into the lungs where they can not be cleared by alveolar macrophages which initiate an inflammatory response leading to lung fibrosis. After phagocytosis of crystalline silica by alveolar macrophages, phagosomes are disrupted realeasing their content into the cytoplasm and producing reactive oxygen species. This result, in the activation of the transcription factor NF*κ*B inducing the gene expression for cytokines including TNF-α, pro-IL-1β and pro-IL-18 and, in the activation of the intracelular sensor known as inflammasome NLRP3 which in turn activates the proinflammatory molecules IL-1β and pro-IL-18. Once the inflammatory stage has started and persists, the fibrotic stage of the lungs appears, where TGF-β plays a central role. TGF-β production is likely induced by citokynes like IL-1β and TNF-α^35^.

Until now, routine clinical tests have not been considered valuable in the diagnosis or follow-up of patients with silicosis. Based on routine clinical analysis of blood samples, we present three biochemical markers that showed differences between controls and patients. The first is ACE, which was previously described to be augmented in patients with classical silicosis^36–39^. Most recently, an elevation of circulating ACE in a group of ES active workers has been reported^40^. Our data in the present work corroborate a progressive increase in circulating levels of ACE only in patients with SS or PMF with respect to HCs, and furthermore, this increase is still maintained for long periods of time (> 6–7 years) after the cessation of exposure. The increase in ACE in the serum of these patients may be generated by immune cells or endothelial cells and be directly related to lung tissue damage^41,42^.

Another molecule that in our analysis showed a significant increase, not only between the healthy group and patients but also between SS and PMF patients, was lactate dehydrogenase (LDH). Although serum LDH is an unspecific marker of cell damage, several studies in humans and in animal models have proposed it as a marker for silicosis^43–48^. More recently, the relation between the plasma level of LDH and the severity of IPF pathology has been described^49^. However, LDH level alterations are observed in many diseases, and this marker on its own should not be considered as a marker to discriminate between different diseases or grades of the diseases.

We also observed a significant increase in plasma soluble fibrinogen values in silicosis patients. Fibrinogen has recently been proposed as one of the markers for silicosis^50^ and could be indicative of the evolution of the fibrotic process in the lungs of silicotic patients, but again, as occurs with ACE and LDH, fibrinogen on its own is not a specific marker for silicosis, and this circulating fibrinogen is elevated in patients with different types of fibrotic diseases^51^.

In our study, plasma C-reactive protein levels, as another marker of the acute phase response, did not present a significant difference when comparing patients and healthy control groups. Accordingly, workers from Swedish iron foundries did not present changes in their circulating CRP blood levels^52^, and neither did patients with different stages of silicosis^28^. However, in other studies including workers exposed to silica, an increase in serum CRP was detected^45,53,54^. Our results for CRP could be explained as a consequence of the fact that the inflammatory state of the patients may have become chronic, rather than acute, due to the time elapsed between exposure to silica, diagnosis and sampling, although further research is needed to confirm this hypothesis.

As we previously commented, although the differences observed in some of the biomarkers analyzed were significant when they were considered individually, they were not discriminative enough for diagnosis and, especially, to differentiate patients belonging to the SS or PMF groups. By analyzing ROC curves evaluating several biochemical biomarkers simultaneously, the stratification of the patients was noticeably improved, allowing fibrinogen-ACE-LDH levels to together discriminate clearly between the HC and SS and PMF groups.

We performed additional analyses to search for simple, rapid and inexpensive clinical biomarkers using white blood cell counts and several related indices that are considered markers of diverse diseases. Recently, some authors^28,29^ have reported that NLR and PLR are related to pulmonary functions and the severity of silicosis in workers exposed to natural silica and can be used as predictors of disease evolution. Here, we have expanded the number of systemic inflammatory indices analyzed and, to our knowledge, this is the first description of leukocyte ratios in patients with ES silicosis. The normal values of our HC group for all the indices presented in Table 2 are in agreement with those published in various studies^55–58^. Then, all the ratios analyzed, NLR, PLR, LMR, SII, SIRI and AISI showed differences between HC and the two groups of patients, which could be especially important in helping to discriminate borderline SS patients from healthy ones. In addition, in the case of LMR, SIRI and AISI ratios, we were able to discriminate between patients with SS and PMF. All these cellular-based inflammatory indices are altered even years after exposure to ES dust has ceased and, therefore, could be markers of an active chronic inflammatory process during the lifelong evolution of the disease, and they might reflect the progression and stage of patients. This needs further study.

Although the AUC values of the ROC curves were acceptable for all biomarkers studied independently, the combination of several variables significantly improved the grouping of the patients according to their disease status. Thus, combining the circulating levels of three simple biomarkers, LDH-ACE-fibrinogen, from routine laboratory tests, we realized good prediction of patient stratification, especially for patients with SS who are borderline according to the ILO classification. The addition of the SIRI index to the combination of the three biochemical markers showed sensitivity and specificity levels nearing 100% in distinguishing PMF patients from healthy controls. However, even though the results combining up to four variables, LDH-ACE-fibrinogen-SIRI, did not present significant differences between SS and PMF patients, the distinction was notably improved when the respiratory test FVC and DLCO were added.

The main limitation of our study is the lack of data on exposure levels during patient activity. We suppose that respirable crystalline silica levels were high because dry processing was common and extractors and other protective measures were not usually implemented^5,10^. Another limitation that has to be mentioned is that we have no validated the algorithm used for multiple indices in an independent group of silicosis patients.

In conclusion, our findings show that a substantial chronic inflammatory state is found in these patients even years after exposure cessation. This could explain the rapid progression of ES silicosis to PMF in a relevant proportion of patients. Therefore, the biomarkers studied here, such as LDH, ACE, fibrinogen and several cellular ratios, such as SIRI, NLR, and PLR, could help to discriminate patients with SS from healthy subjects and even provide an additional element to outline patient profiles together with radiological and respiratory function tests, and could predict the severity of the disease. Additionally, all these biomarkers are readily available and can be obtained from routine blood tests that are usually requested as part of routine care. Further research is needed with longitudinal studies that clarify the role of these biomarkers to identify patients who will evolve to PMF and the specific role of these ratios in discriminating between healthy workers and SS or PMF patients.

## Materials and methods

### Study subjects

All patients included were male workers who were working in cutting, polishing and finishing of engineered stone countertops. They are part of a cohort of patients followed by the Pneumology, Allergy and Thoracic Surgery Department of Puerta del Mar University Hospital in Cádiz (Spain). Patients were diagnosed with silicosis based upon a history of exposure to silica and chest radiography and/or HRCT and, in some cases, by lung or mediastinal lymph node biopsy. Chest radiographs and HRCT scan classification and progression of these patients have been described previously^10^. Those patients, in follow-up in our outpatient clinic, were asked to take part in the prospective phase of the project approved by the institutional Research Ethics Committee of the province of Cadiz on December 20, 2016, adding a blood draw for laboratory tests to the standard procedures (respiratory function tests and chest radiography). From this initial cohort (106 patients), 79 of them accepted to participate in the prospective phase. Five patients had died (two of them after lung transplantation and three for reasons unrelated to silicosis). The rest of the patients were lost in the follow-up or did not accept to participate. In October 2018 we started a new project approved by the institutional Research Ethics Committee of the province of Cadiz that included newly recruited patients and those from the original cohort that accepted the follow-up. This manuscript presents the data from the analysis of the blood sample of each patient from a cohort taken from June 2017 to June 2020. Two specialized pulmonologists classified patients into SS or PMF attending to radiologic criteria^13,14^. The exclusion criteria for a patient from the study were active infection, kidney or liver disease, autoimmune rheumatic disease, or current use of immunosuppressive drugs; only oral corticosteroids at a dose lower than 20 mg per day were accepted.

Respiratory function tests were performed by trained personnel using a Master Screen PFT/Body System (Jaeger, Viasys, CareFusion) on the same day of blood extraction for biochemical and hematological tests. The data collected included forced vital capacity (FVC), forced expiratory volume in 1 s (FEV1), the ratio of FEV1/FVC and diffusing capacity of lung for carbon monoxide (DLCO) measured by the single-breath procedure following international recommendations^10,59,60^.

Twenty-two male volunteers with no history of exposure to silica dust were used as the healthy control (HC) group. All of them were hospital staff workers and none of them had respiratory symptoms or chronic or acute disease. The medical evaluation, prior to blood sampling, was normal in all cases.

This study was conducted according to the guidelines of the Declaration of Helsinki, and approved by the institutional Research Ethics Committee of the province of Cadiz (registration n° 90.18, date 29/09/2018). The SSPA Biobank of the Hospital Universitario Puerta del Mar (HUPM, Cádiz, Spain) coordinated the collection, processing and management of samples and clinical data according to the standard procedures established for this purpose. Informed consent was obtained from all participants involved in the study.

### Biochemical and hematological analyses

Overnight-fasting blood samples were collected in EDTA tubes and processed immediately for general biochemistry and hematological analysis. These were performed by the analysis unit of Puerta del Mar University Hospital and included fibrinogen (mg/dL) using the ACL-TOP® CTS300 analysis system (Werfen, Spain), C-reactive protein (mg/L), lactate dehydrogenase (IU/mL) using the Alinity™ analysis system (Abbot, Spain) and angiotensin-converting enzyme (IU/L) using the BA-200 analysis system (Biosystem, Spain). Leukocyte populations were analyzed using an automated XN-1000 hematology analyzer (Sysmex, Germany), and the NLR, PLR, LMR SII, SIRI and AISI were calculated.

### Statistical analysis

SPSS software (IBM Statistics) was used for statistical analysis. Initially, the normality distribution of every data set was established using the Kolmogorov–Smirnov test. Subsequently, one-way ANOVA for multiple (generally three: HC, SS, PMF) groups of data was performed by the ANOVA F-test (normal distribution) or by the Kruskal–Wallis test (non-normal distribution). For comparisons of two groups of data (HC vs. SS, HC vs. PMF or SS vs. PMF), Student’s t-test (for normally distributed data) or the Mann–Whitney U-test (for non-normally distributed data) was used. The chi-square statistic was used to test relationships between categorical variables. The results are expressed as the mean and SD. A significance level of *p* ≤ 0.05 was adopted for all tests.

Receiver operating characteristic (ROC) curves were generated considering HC as the reference group in the HC vs. SS and HC vs. PMF comparisons and using SS as the reference group in the SS vs. PMF comparison. Multiple logistic regression analyses were performed for combined ROC curves. The regression was calculated by the interception, main effects and two-way interactions between two groups (HC vs. SS; HC vs. PMF; SS vs. PMF) and using the corrected Akaike information criterion.

## Supplementary Information

Supplementary Information.

## Data Availability

Existing ethical permits do not allow that personal data from this study are deposited in the public domain. The full dataset is available for researchers who meet the criteria for confidential data access as stipulated by participant informed consent and the Institutional Research Ethics Committee of the province of Cadiz (registration n° 90.18, date 29/09/2018), Spain.
