# Supplementary material for: Inflammatory indices obtained from routine blood tests show an inflammatory state associated with disease progression in engineered stone silicosis patients
Source: Sci Rep. 2022 May 17;12:8211. doi: 10.1038/s41598-022-11926-x (PMC9114118; doi:10.1038/s41598-022-11926-x)
Supplement: Supplementary file 1 — Supplementary Information. [file 41598_2022_11926_MOESM1_ESM.pdf]

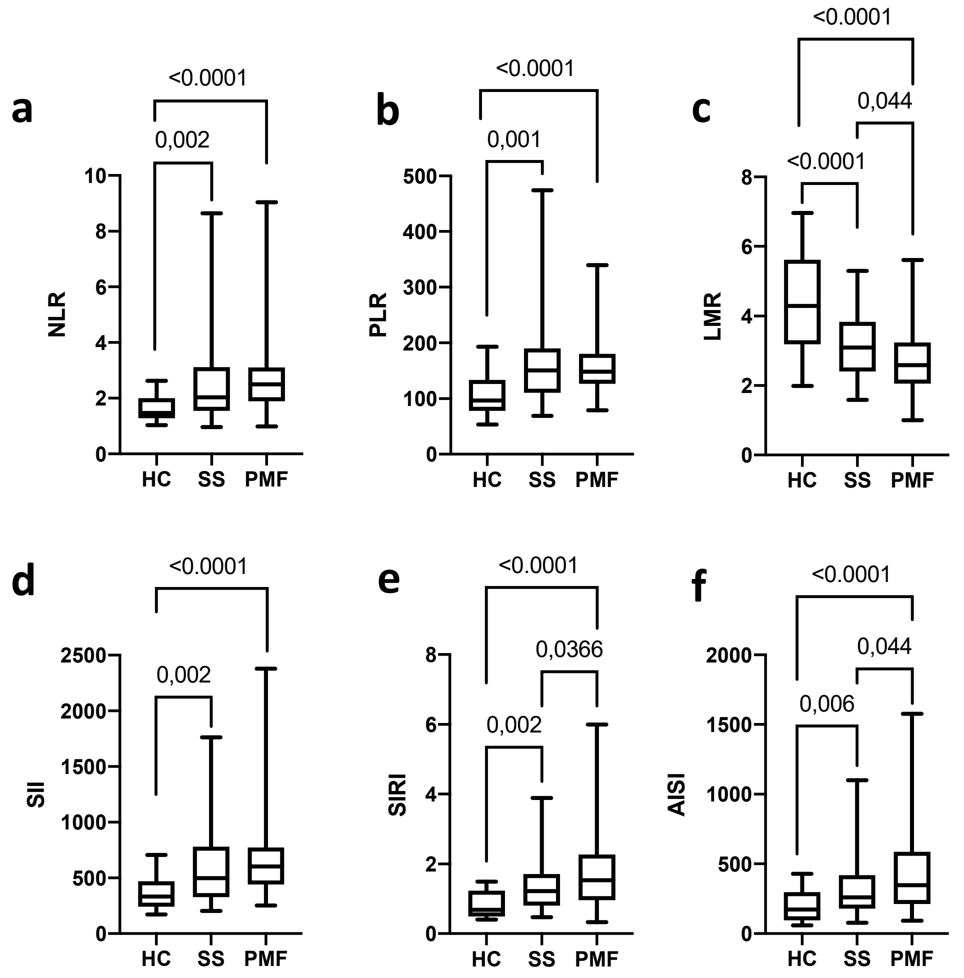

Figure S1. Box plots showing results of leukocytes subset indices from Table 2. (**a**) NLR (neutrophil/lymphocyte ratio), (**b**) PLR (platelet/lymphocyte ratio), (**c**) LMR (lymphocyte/monocyte ratio), (**d**) SII (neutrophil x platelet/lymphocyte ratio), (**e**) SIRI (neutrophil x monocyte/lymphocyte ratio), (**f**) AISI (neutrophil x monocyte x platelet/lymphocyte ratio). Healthy Control (HC), Simple Silicosis (SS) and Progressive Massive Fibrosis (PMF) Boxes show interquartile ranges. Lines in the boxes correspond to medians, and bars represent the lowest and highest values. *p* values with significant differences between groups are indicated by horizontal lines.
